# Perceptual Asymmetries and Auditory Processing of Estonian Quantities

**DOI:** 10.3389/fnhum.2021.612617

**Published:** 2021-04-28

**Authors:** Liis Kask, Nele Põldver, Pärtel Lippus, Kairi Kreegipuu

**Affiliations:** ^1^Institute of Psychology, University of Tartu, Tartu, Estonia; ^2^Doctoral School of Behavioural, Social and Health Sciences, University of Tartu, Tartu, Estonia; ^3^Institute of Estonian and General Linguistics, University of Tartu, Tartu, Estonia

**Keywords:** auditory perceptual asymmetry, mismatch negativity, speech perception, duration change, pitch change, quantity stimuli, language processing

## Abstract

Similar to visual perception, auditory perception also has a clearly described “pop-out” effect, where an element with some extra feature is easier to detect among elements without an extra feature. This phenomenon is better known as auditory perceptual asymmetry. We investigated such asymmetry between shorter or longer duration, and level or falling of pitch of linguistic stimuli that carry a meaning in one language (Estonian), but not in another (Russian). For the mismatch negativity (MMN) experiment, we created four different types of stimuli by modifying the duration of the first vowel [ɑ] (170, 290 ms) and pitch contour (level vs. falling pitch) of the stimuli words (‘SATA,’ ‘SAKI’). The stimuli were synthesized from Estonian words (‘SATA,’ ‘SAKI’) and follow the Estonian language three-way quantity system, which incorporates tonal features (falling pitch contour) together with temporal patterns. This made the meaning of the word dependent on the combination of both features and allows us to compare the relative contribution of duration and pitch contour in discrimination of language stimuli in the brain via MMN generation. The participants of the experiment were 12 Russian native speakers with little or no experience in Estonian and living in Estonia short-term, and 12 Estonian native speakers (age 18–27 years). We found that participants’ perception of the linguistic stimuli differed not only according to the physical features but also according to their native language, confirming that the meaning of the word interferes with the early automatic processing of phonological features. The GAMM and ANOVA analysis of the reversed design results showed that the deviant with longer duration among shorter standards elicited a MMN response with greater amplitude than the short deviant among long standards, while changes in pitch contour (falling vs. level pitch) produced neither strong MMN nor asymmetry. Thus, we demonstrate the effect of language background on asymmetric perception of linguistic stimuli that aligns with those of previous studies (Jaramillo et al., 2000), and contributes to the growing body of knowledge supporting auditory perceptual asymmetry.

## Introduction

When perceiving the input from the outside world, an element with something extra (e.g., visually presented letter Q) is usually easier to detect among elements without the feature (e.g., letter O) than a lesser element (O) among elements with those extra features (Q) (Treisman, 1985; Wolfe, 1994). This phenomenon has been described in different sensory modalities, including auditory modality, where such discrepancies are known as auditory perceptual asymmetry. The basis for the theoretical background of auditory perceptual asymmetry has been proposed but it is not fully clear if and how language experience can affect the asymmetry for linguistic stimuli. Brain imaging techniques, such as electroencephalography (EEG), and more precisely mismatch negativity (MMN), allow us to study these effects with great temporal resolution.

### Mismatch Negativity (MMN)

Mismatch negativity is a pre-attentive response in the brain to stimuli that are rare or deviant among frequently presented standard stimuli (Näätänen et al., 2007). The MMN is the subtraction of the averaged event-related potentials (ERP) evoked by the standard stimulus from the averaged ERPs evoked by the deviant stimulus. The key element of the MMN generation is building an internal model or memory trace for the standard stimulus (Näätänen, 2001; Sussman, 2007). This model forms a basis against which the next incoming stimuli are compared to. If the new input matches the model, sparing of processing resources can be achieved by stimulus-specific adaptation and ease of memory-comparison (due to familiarity). If it does not match, new firing units and “surprise” from a memory-comparison evoke processing that is known as the MMN. MMN depends critically on the size of the difference between the standard and the deviant stimulus, and the ease for the brain to build the internal model of the standard stimulus. Thus, the MMN relies on the predictive coding paradigm – the brain learns from the input, constantly generates predictions for possible future outcomes and recognizes discrepancies based on these expectations (Garrido et al., 2009; Scharinger et al., 2012).

### Auditory Perceptual Asymmetry and MMN

Auditory perceptual asymmetry can be easily operationalized through MMN. The standard stimulus and the deviant stimulus may reverse their roles (i.e., X is the standard and Y is the deviant in one series, while Y is the standard and X is the deviant in another series). The subtracted ERPs for Y-X and X-Y in respective series directly reflect how much Y differs perceptually from X and vice versa, X from Y. Early reports show the symmetry between both change directions in non-linguistic sounds (Nordby et al., 1988; Näätänen et al., 1989), but there are also reports showing clear asymmetric tendencies (Takegata et al., 2008).

The way auditory stimuli are processed can be inferred from the (a)symmetry of the reversed MMN responses. Timm et al. (2011) propose two hypotheses on how differences in sounds can be detected: the information-content hypothesis (Sinkkonen et al., 1996; Sinkkonen, 1999) and the feature-detector hypothesis (Bishop et al., 2005). The information-content hypothesis focuses on the difference between novelty (the expected probability) of standard and deviant sounds that determines the allocation of processing resources resulting in the symmetrical MMN responses even when the stimuli have reversed positions. On the other hand, the feature-detector hypothesis supports asymmetric processing – detecting the difference in deviant sounds is dependent upon additional features that appear in the deviant but not in the standard. As such, adding a feature to a deviant should enhance the MMN as the activity of feature detectors increases. In their study with sine wave stimuli, Timm et al. (2011) found support for the feature-detector hypothesis and noted similar result patterns a number of previous studies (Nordby et al., 1994; Sabri and Campbell, 2000; Bishop et al., 2005).

### The Role of Long-Term Language Experience

Linguistic stimuli are a special case of auditory stimuli as in their case, the brain makes linguistic predictions based not solely on the sensory attributes (like intensity, frequency or duration, e.g., Näätänen et al., 2004) but also on the phonological knowledge of a native language (Kuhl, 2004; Gagnepain et al., 2012). Both, low-level sensory attributes and phonological knowledge about categories in language, may help in forming features (c.f., Bishop et al., 2005; Timm et al., 2011; Schluter et al., 2016). The phonological information of one’s own native language and semantic processing are important as they “sharpen the ear” and facilitate model-building. In principle, it can be expected that attributes that are presented in one’s native language are better discriminated due to better memory traces and accessibility, long-term tuning and perceptual learning (Gibson and Gibson, 1955). Information encoded into linguistic categories and sound contrasts (the functional significance of linguistic categories helping to store information in the mental lexicon) can affect perceptual processes (Kazanina et al., 2006). This influences auditory asymmetry – features that are present within a language (e.g., pitch contour or vowel duration that can change a word’s meaning) and therefore have created a strong long-term memory trace, determine how precisely our auditory perception decodes and compares each element of the sound.

This is also supported by observations with asymmetric effects when both (standard and deviant) stimuli are meaningful words but differ in their frequency in a particular language. As less familiar words get a limited number of repetitions in the mental lexicon, it is harder to recognize these as meaningful words and distinguish them from pseudo words (Aleksandrov et al., 2017a). The MMN responses to well-known (high-frequency) words have greater amplitudes and earlier latencies compared to those of less-known (low-frequency) words (Davis and Gaskell, 2009; Tamminen et al., 2015; Aleksandrov et al., 2017a). The MMN response to pseudo words also differs from real words (Shtyrov et al., 2005; Shtyrov and Pulvermüller, 2007) as the main differences are between acoustic or structural traits, and the processing is not influenced by the meaning (Pulvermüller et al., 2001; Aleksandrov et al., 2017b), generating a MMN with longer duration, later latency and lower amplitude.

### Auditory Perceptual Asymmetry of Linguistic Stimuli

As noted in the previous section, in addition to the magnitude of physical differences between stimuli under comparison, consistency of the linguistic representation (i.e., underspecification, Lahiri and Reetz, 2002 or prototypicality, Ikeda et al., 2002) may also play a role in perceptual asymmetry. Underspecification of lexical features (Lahiri and Reetz, 2002) is a mechanism through what the asymmetry may appear. It refers to the idea that from all possible phonological features of a particular sound not everything is stored in or is not an essential part of a mental representation of the linguistic category this sound belongs to. These features are underspecified in that the model resulting in a weaker MMN. For instance, Eulitz and Lahiri (2004) have shown that MMN to phonemic contrast is asymmetric to a phoneme pair when one of the phonemes is underspecified compared to the other. Similar asymmetry was demonstrated with Mandarin (Li and Chen, 2015; Politzer-Ahles et al., 2016) and Cantonese (Law et al., 2013) tones. Politzer-Ahles et al. (2016) pointed out that previous studies have concentrated chiefly on only one language group, and adding comparisons with language naïve listeners and cross-linguistic research can add additional value to these studies. Regarding prototypicality (Ikeda et al., 2002), an earlier or larger MMN is elicited when the standard is a good representative of the category. It happens because when the match between the standard stimulus and the linguistic category it represents is high, the mental model against which subsequent deviant stimuli are compared to is more easily formed.

When talking about a feature in case of linguistic stimuli, it should not be understood too narrowly as it could either be part of a linguistic category as well as refer to low-level processing of language stimuli (c.f., Bishop et al., 2005; Timm et al., 2011; Schluter et al., 2016). For linguistic stimuli, the two meanings of a feature are interwoven and the MMN inevitably includes both low-level and high-level processing. In the current study, we concentrate on the phonetic features that are important in Estonian language – duration and pitch. As vowel duration and pitch contour in language stimuli are known to be discriminated according to the Weber’s law, i.e., relative to a standard (e.g., Lehiste, 1970), a long deviant represents a greater change when compared to a short standard than a short deviant compared to a long standard. Similarly, in case of pitch contour, detecting a pitch change (i.e., falling pitch as a deviant among level pitch as a standard) probably results in a bigger difference (i.e., an earlier or larger MMN) than a non-change detection (i.e., level pitch as a deviant among falling pitch as a standard). The direction of the assumed asymmetry, in case of a pitch change, may also be facilitated by neural fatigue (and is reflected by the N1 component, Näätänen et al., 2005) as any new information is encoded by fresh neural units and thus, result in a greater MMN. Long-term experience with one language can alter the importance of specific linguistic characteristics and through that add an additional layer of complexity to already discussed asymmetric effects in auditory perception. Consequently, within a MMN design, asymmetry is likewise expected to be greater for native-language-compatible attributes, which is observed in the current study.

### Current Study: Estonian and Russian Language Compared

We focus on the (a)symmetric detection of duration and pitch changes in Estonian language stimuli in two different groups of participants: native Estonian-speakers and native Russian-speakers. The Estonian language, belonging to the Finno-Ugric language family, is a quantity language. The duration of speech sounds is contrastive at the lexical level where it can affect word meaning. Estonian is known for its complex and typologically rare three-way quantity system. The three length degrees are manifested primarily by the duration of the vowel or the final consonant of the stressed syllable: short (Q1) sagi [sɑ.ki] ‘bustle, imp.sg2’; long (Q2) saagi [sɑ:.ki] ‘harvest, gen.sg,’ saki [sɑk.ki] ‘tab, gen.sg’; overlong (Q3) saagi [sɑ::.ki] ‘saw, part.sg,’ sakki [sɑk:.ki] ‘tab, part.sg’. Phonetically this distinction is realized by combinations of segmental duration and tonal patterns (Lehiste, 1997; Lippus et al., 2007, 2009, 2013). The domain of the quantity is the trochaic i.e., disyllabic left-headed foot. The duration of the unstressed vowel shortens as the stressed syllable gets longer and the quantity is perceived from the duration ratio of the segments within the foot (Lehiste, 1997; Traunmüller and Krull, 2003). The contrast between Q1 and Q2 vs. Q3 is supplemented by the pitch contour. The high level pitch falls at the end of the stressed syllable in Q1 and Q2 but already in the first half of the stressed syllable in Q3 (Lehiste, 1997; Lippus et al., 2013), making this early peak alignment an important cue for perceiving Q3 (Lehiste, 1997; Lippus et al., 2009). The Russian language, does not follow a similar structure concerning duration and pitch contour (Bondarko, 1977), making it relatively difficult for Russian native speakers to differentiate Estonian quantities.

### Duration and Auditory Perceptual Asymmetry

Duration can change a word’s meaning in many different languages, including Estonian. Although Russian does not have phonologicallength category, lexical stress has a comparable significance as itis mainly associated with the duration of a phoneme (Bondarko, 1977, as cited in Meister, 2011), for example ‘Mýκa’ – ‘agony’, ‘Myκá’ – ‘flour’ (Hint, 1998). Studies have shown that a decrease in duration can be harder for the brain to detect than an increase. Jaramillo et al. (2000) viewed a smaller MMN amplitude when the deviants were shortened compared to the standards, while longer deviants (compared to the short standard) elicited a bigger MMN response. Roberts et al. (2014) compared non-word mispronunciations of spoken words which had different durations of a medial consonant and found asymmetric responses to duration changes as an increased duration did not impede lexical access while a decrease in duration weakened the response. A significant difference in the direction of deviance has also been presented by Peter et al. (2010) – the increment MMN was more stable than the decrement MMN. Still, some studies have not always supported the same conclusions or have even proven the opposite, as the elicited MMN has been found to be larger for short deviants compared to the long ones (Colin et al., 2009).

Based on the previously discussed studies, we expect durational differences to be easier to detect (compared to pitch) for both language groups, Estonians and Russians, as duration is involved in lexical contrasts in both languages, and as duration as a linguistic feature is physically more easily distinguishable (having a clear cut-off point). A short duration of the standard stimulus (compared to the long deviant) should give rise to asymmetric MMNs for both language groups. Importantly, the short duration of the stimulus (with the first vowel 170 ms) used in the current study is linguistically close to the long quantity (Q2) in Estonian. This means that the stimulus duration determines the linguistic meaning here (and that deviations from the Q2 fall easily either to Q1 or Q3), and this short duration could be considered a default feature for the Estonian subjects. The asymmetry between the increment and decrement MMNs is expected to be bigger for the native (Estonian) speakers due to their language background, and the fact that the current study implements Estonian language specific stimuli that are meaningful words only to the Estonian participants.

### Pitch and Auditory Perceptual Asymmetry

Tonal features are hard to learn for non-native speakers if their native language does not use similar pitch contrasts, as the brain loses the ability to distinguish non-native phonemes and structural components after the active period of language development (Dupoux and Peperkamp, 2002; Best, 2019; Gosselke Berthelsen et al., 2020). In such situations, non-native speakers may start to use different cues than those used by native speakers (Stragay and Downs, 1993). Liu et al. (2018) demonstrated how the MMN to non-native tonal contrast was dependent upon learning and the direction of the change – the participants who heard a falling tone deviant in the first experimental block had larger MMN response than the ones who first heard the level tone deviant.

For the perception of contrastive word stress, Russian-native listeners mainly use duration and the intensity cue while ignoring vowel quantity and pitch (Chrabaszcz et al., 2014). For example, Russian native speakers tend to rely only on durational cues even while discriminating Estonian Q2 and Q3 while Estonians use pitch cues (Lippus et al., 2009; Meister, 2011; Chrabaszcz et al., 2014). It is possible that because of their own language structure, Russian native speakers do not perceive the change in pitch as clearly, and are not able to distinguish quantities by using changes in duration alone, making it difficult for them to choose the correct quantity. Level pitch can be considered as the default pitch contour in Estonian language (Lippus et al., 2013) as it is typical for the short (Q1) and long (Q2) quantity degrees while the falling pitch is the secondary cue for the overlong quantity (Q3). Changes in pitch are specific to Estonian and thus carry an importance mainly for Estonian native speakers, which may be elucidated through MMN. In this study we hypothesize that pitch will be harder to distinguish for Russian native speakers who have previously had limited contact with pitch languages, as it is not an important auditory feature for them. Accordingly, we expect both, a bigger MMN amplitude and bigger asymmetry, appear among Estonian native speakers as their language experience has created an advantage to perceive the difference in pitch contour, and as the stimuli are meaningful words for them.

### Current Study: Hypotheses

We compare two pairs of stimuli [(1) short deviant and long standard vs. long deviant and short standard, (2) deviant with falling pitch and standard with level pitch vs. deviant with level pitch and standard with falling pitch] in reversed positions in order to explore perceptual asymmetry in Estonian and Russian native speakers. The two groups help us investigate the possible differences in perceptual asymmetry for duration and pitch when the stimuli are meaningful words for one group (Estonian native speakers) and pseudo words for another (Russian native speakers), or when the physical features (duration, pitch) belong or do not belong to one’s native language.

We set the following hypothesis:

**H1: The discrimination of the deviant and standard stimuli is asymmetrical:**

H1.1.: A MMN response is earlier or with a larger amplitude for the longer duration deviant among shorter standards than short deviant among long standards;
H1.2.: A MMN response is earlier or with a larger amplitude for the deviant with falling pitch among standards with level pitch than the deviant with level pitch among standards with falling pitch.

**H2: The asymmetrical discrimination between the deviant and standard stimuli depends on long-term language experience (native language):**

H2.1.: Estonian native speakers have a similar asymmetric MMN response for both type of stimulus change (tonal and durational change);
H2.2.: Russian native speakers MMN amplitude and onset is influenced by asymmetric effects only in conditions with durational change.

Irrespective of asymmetry, we expect the MMNs for Estonian native speakers to be more pronounced than that of Russian native speakers.

## Materials and Methods

### Participants

Twenty-four volunteers (18–27 years old) participated in the study. Twelve of the participants were native Russian speakers (10 female, 2 male) (mean age 23.4, *SD* = 2.90) and 12 native Estonian speakers (10 female, 2 male) (mean age 23.7, *SD* = 2.80). Participants from both groups were matched with each other by gender and age. All participants had normal hearing (checked before the experiment with an audiometer), no serious psychiatric or neurological conditions, and normal or corrected-to-normal eyesight. All but one participant were right-handed. Two native Russian speakers had a second native language (Ukrainian and Karachay-Balkar) that structurally differed from Estonian significantly, and including their data did not alter the results. Russian speakers were foreign students at the University of Tartu, who had been in Estonia for a limited time period (4.4 months on average). All Russian participants had passive contact with Estonian through their studies and all were temporarily living in Estonia at the time of the experiment. Nine Russian participants reported having no Estonian language skills. Three participants who had already attended Estonian language classes reported passive language skills (*I can understand the basics and speak some of the language*) but none of them reported using Estonian for socializing or spent extensive time in an Estonian language environment. The influence of time spent in Estonia and the aforementioned language experiences were controlled for, and they had no significant effect on the results.

The study presented here was approved by the Research Ethics Committee of the University of Tartu [based on The Code of Ethics of the World Medical Association (Declaration of Helsinki)], and all participants provided written consent. The experimental procedure was introduced to the participants before the beginning of the experiment, and they had the option to stop the experiment at any point in time.

### Study Design

Before the experiment, all participants filled out an online background questionnaire (via a web-based research portal of the Institute of Psychology, University of Tartu), asking about birthplace, education, language skills, relevant medical conditions, musicality, and handedness. Russian native speakers had additional questions about the time spent in Estonia and their prior experience with the Estonian language. The testing procedure included an audiometric measurement, Estonian Words in Noise (EWIN) speech intelligibility test (Veispak et al., 2015), pre- and post-experiment critical flicker frequency test (CFF, Simonson and Brožek, 1952), an adapted Borg CR-10 scale (Borg, 1998) before, after, and three times throughout the experiment to measure subjective fatigue, a subjective scale to register the mood, and an EEG experiment itself (lasted for about 1.5 h). Only the results of the EEG experiment are presented in the current paper.

### Stimuli: Description and Presentation

The stimuli used within this study were synthesized from Estonian words from the two sound sequences ‘SATA’ and ‘SAKI’ (see Table 1). It is important to note that even though all stimuli represent a full meaningful word in Estonian, the words in the ‘SATA’ sequence are more frequent in Estonian than the words in the ‘SAKI’ sequence (‘SATA’ different forms are among 1000 most frequent word forms and among 10,000 most frequent lemmas, no variation of ‘SAKI’ belongs among most frequent words or lemmas; Frequency lists in The Balanced Corpus of Estonian^1^). The stimuli were generated with the program Praat (Boersma and Weenink, 2007) by re-synthesizing the recordings of naturally read speech produced by a male native Estonian speaker. From each of the recorded words, a set of nine stimuli were created by manipulating the duration of the stressed vowel of the recorded words in steps of 30 milliseconds. The full set of stimuli of the ‘SATA’ sequence had been used before in a behavioral study (by Lippus et al., 2009) and the ‘SAKI’ stimuli set was created to match the physical properties of the former to expand the generalizability of possible effects.

**TABLE 1:** Stimuli words and their meaning in Estonian.

Quantity	Stimulus set ‘SAKI’	Stimulus set ‘SATA’
Short— Q1	*sagi* [sɑ.ki] ‘bustle, imp.sg2’	*sada* [sɑ.tɑ] ‘hundred, nom.sg’
Long – Q2	*saagi* [sɑ:.ki] ‘harvest, gen.sg’	*saada* [sɑ:.tɑ] ‘send!, imp.sg’
Overlong – Q3	*saagi* [sɑ:.ki] ‘saw, part.sg’	*saada* [sɑ:.tɑ] ‘to get, imp.sg’

**FIGURE 1:**
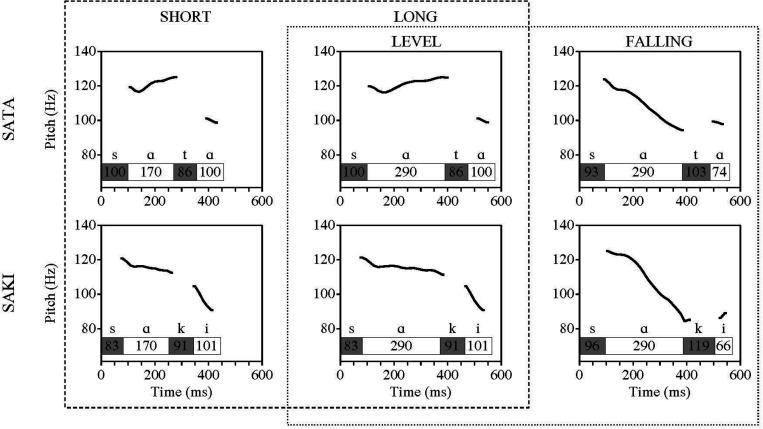
Overview of stimuli used (duration of syllables and pitch contour). The specifications of the first vowel are marked on a row above the columns. The length of the first vowel was either 170 or 290 ms, and had either a level or falling pitch contour. The length of other consonants and vowels was fixed for every stimuli – first consonant (S) 100 ms, second consonant (T/K) 86 ms and second vowel (A/I) 101 ms. Dashed line marks comparisons with duration change, dotted line marks comparisons with changes in pitch contour.

Based on the results of the Lippus et al. (2009) behavioral study, four different stimuli were selected from both ‘SATA’ and ‘SAKI’ word sets. These were acting as standard or deviant stimuli presented in the optimal MMN paradigm (Näätänen et al., 2004). There were four experimental series: in each, one of the four stimuli acted as a repeating standard and the other three as intermittent deviants. For testing the hypotheses of the current study and to examine the possible asymmetry effects, we used three stimuli out of three experimental series (illustrated in Figure 1):

(1) Level pitch and shorter first vowel (V1) duration. The stimulus was derived from a long quantity (Q2) word with properties of a typical Q2 word and perceived as representing Q2 by 99% of responders of the behavioral study;
(2) Level pitch and long V1 duration. The stimulus was derived from the same Q2 word, V1 duration typical for Q3 word but tone typical for Q2, perceived as Q2 by 55% of responders;
(3) Falling pitch and long V1 duration. The stimulus was derived from an overlong (Q3) word, carrying the properties typical for Q3 word, and perceived as Q3 by 95% of the responders (Lippus et al., 2009).

The three stimuli were combined into pairs for comparison (Table 2). The paired stimuli differed from each other by the manipulation of a single feature (duration or pitch change) and can be used for a reversed analysis design without unexpected confounding factors: in two, the pairs consisted of short and long stimulus with level pitch; in the other two, the pairs consisted of long stimuli with level and falling pitch. The fourth stimulus (and consequently the fourth series where it acted as a standard) was left out of the analyses, because it carried two feature manipulations (pitch as well as a very short duration).

**TABLE 2:** Description of the stimulus pairs used for comparison.

	Short, Level	Long, Level	Long, Falling
Pair 1	**STANDARD**	DEVIANT	–
Pair 2	DEVIANT	**STANDARD**	–
Pair 3	–	**STANDARD**	DEVIANT
Pair 4	–	DEVIANT	**STANDARD**

In each series, the number of deviants was 100, and the number of standards was 315. For the analysis, the number of standards was equalized to the number of deviants they were compared to by selecting the standard stimuli that were presented immediately before the respective deviants. The interstimulus interval (ISI) was 400, 425, or 450 ms. Different lengths of ISI were used as it resembles natural speech and prevents conditioning of the coming response.

### EEG Recording and Procedure

The EEG experiment (64-electrode ActiveTwo system, BioSemi B.V., Amsterdam, Netherlands) consisted of four series, each lasting for 11 min, and additional pre- and post-experiment resting state EEG recordings. Two reference electrodes were attached to the earlobes, and four single electrodes to record eye-movements and blinks were attached to the participants face, close to the eyes. The earlobe reference electrodes were linked in post-processing. The EEG data were recorded using a 512 Hz frequency and 0.6–100 Hz filters. The auditory stimuli were presented to the headphones with custom MATLAB (MathWorks, Natick, MA, United States) programs, always with the same volume not exceeding 60 dB HL (hearing level). To distract their attention away from the presented sounds, the participants watched a soundless cartoon on the Mitsubishi Diamond Pro 2070SB 22′′ computer screen (Mitsubishi Electric, Tokyo, Japan) (participant’s chair was approximately 114 cm away from the screen).

### EEG Data Analyses

For EEG offline analysis, we used Brain Vision Analyzer 2.1 (Brain Products GmbH, Munich, Germany). A Butterworth Zero Phase Filter (0.1–30 Hz, 24 dB/oct) was used to reduce noise, and the Gratton and Coles algorithm (Gratton et al., 1983) was used to reduce the influence of eye-movements and blinks. Segments of EEG were chosen and separated from the main dataset for analysis (from 100 ms before stimulus onset to 600 ms after). Baseline correction was done 100 ms before stimulus onset, and the following artifact removal criteria were used: 50 μV as the maximum allowed voltage step, –75 and 75 μV as the minimum and maximum permitted amplitudes, and 0.5 μV as the lowest activity in an interval of 100 ms. For the ERPs, the signals were individually averaged for every stimulus in every series, and accordingly the individual MMN difference waves (standard minus deviant) were found. Data for each stimulus and the MMN for each series were then averaged together across participants.

Temporal and frontal scalp areas were chosen as areas of interest based on previous research (see Kujala et al., 2007 for a review), and four electrodes with relatively well-detectable MMN activity [AF3 (left frontal), AF4 (right frontal), C3 (left temporal), C4 (right temporal)] were chosen and included into the analysis. Generalized Additive Mixed Models (GAMM) were run on single standard and deviant trials (–100.600 ms) of all participants, stimuli and series under comparison. Four time intervals (400–420, 420–440, 440–460, 460–480 ms) were included in the later analyses and were selected through inspection of the peaks of deviant activity and the results of GAMM analysis. When looking at the standard and deviant (and subsequent MMN) activity, the first 100 ms (for ‘SATA’) or 83 ms (for ‘SAKI’) should be subtracted from the latency values, as this is the time when the first vowel (incorporating the duration or pitch changes manipulations) appeared, and therefore not important in the context of the research question of this paper (see Figure 1). The set-point where the standard and deviant difference (i.e., the MMN) was expected to start depends on the duration of first vowel and the start of the physical difference of the two stimuli (around 170 ms).

Organization and analysis of raw data was carried out using R (R Core Team, 2020) and RStudio (RStudio Team, 2015). Packages mgcv (Wood, 2006) and itsadug (Rij et al., 2020) were used for GAMM, ez (Lawrence, 2016) was used for repeated measures ANOVAs, simpleboot (Peng, 2019) and MKinfer (Kohl, 2020) for bootstrap analyses. *Post hoc* analyses were conducted using the Bonferroni HSD test.

## Results

**FIGURE 2:**
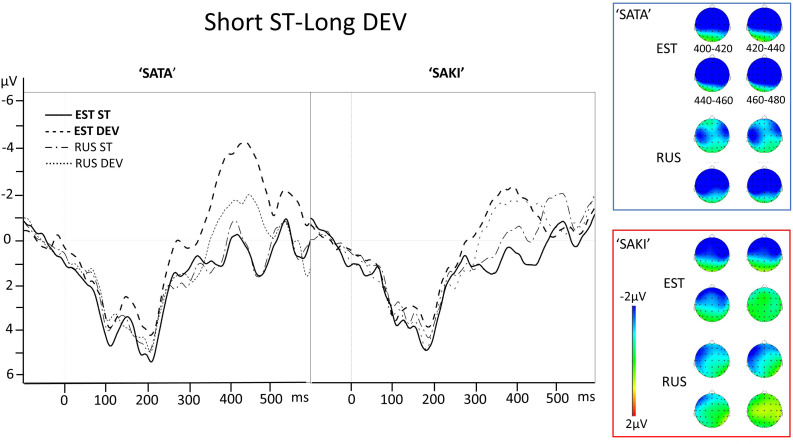
Electrode AF3 averaged ERP activity for standard (ST) and deviant (DEV) stimuli in ‘Short ST-Long DEV’ comparisons for ‘SATA’ and ‘SAKI’ word set in Estonian (EST) and Russian (RUS) Language groups. Right panel represents distribution of averaged mismatch response (DEV-ST) in 400–480 ms. Please observe, that first 83–100 ms are exactly the same for ST and DEV under comparison (consonant, see stimuli in Figure 1).

**FIGURE 3:**
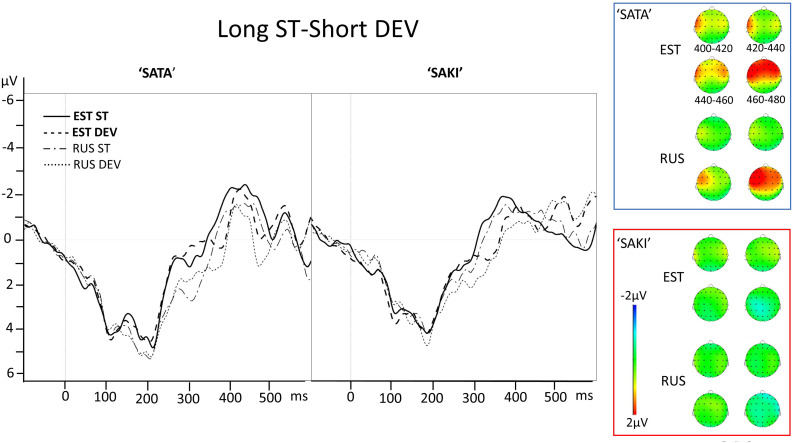
Electrode AF3 averaged ERP activity for standard (ST) and deviant (DEV) stimuli in ‘Long ST-Short DEV’ comparisons for ‘SATA’ and ‘SAKI’ stimuli in Estonian (EST) and Russian (RUS) Language groups. Right panel represents distribution of averaged mismatch response (DEV-ST) in 400–480 ms. Please observe, that first 83–100 ms are exactly the same for ST and DEV under comparison (consonant, see stimuli in Figure 1).

**FIGURE 4:**
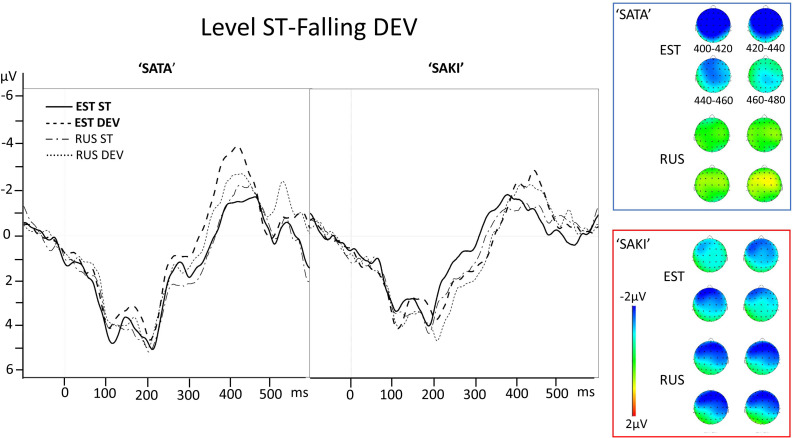
Electrode AF3 averaged ERP activity for standard (ST) and deviant (DEV) stimuli in ‘Level ST-Falling DEV’ comparisons for ‘SATA’ and ‘SAKI’ stimuli in Estonian (EST) and Russian (RUS) Language groups. Right panel represents distribution of the averaged mismatch response (DEV-ST) in 400–480 ms. Please observe, that first 83–100 ms are exactly the same for ST and DEV under comparison (consonant, see stimuli in Figure 1).

**FIGURE 5:**
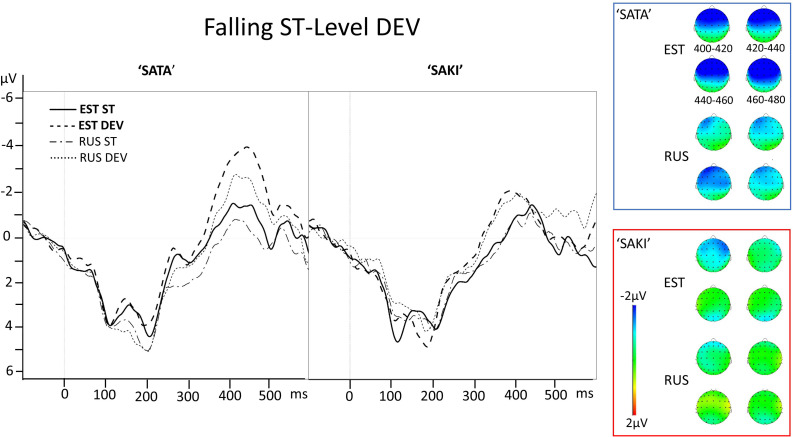
Electrode AF3 averaged ERP activity for standard (ST) and deviant (DEV) stimuli in ‘Falling ST-Level DEV’ comparisons for ‘SATA’ and ‘SAKI’ stimuli in Estonian (EST) and Russian (RUS) Language groups. Right panel represents distribution of averaged mismatch response (DEV-ST) in 400–480 ms. Please observe, that first 83–100 ms are exactly the same for ST and DEV under comparison (consonant, see stimuli in Figure 1).

First the averaged waveforms of standard (ST) and deviant (DEV) stimuli for each Word set (‘SATA,’ ‘SAKI’) and Comparison (‘Short ST-Long DEV’ vs. ‘Long ST-Short DEV,’ ‘Level ST-Falling DEV’ vs. ‘Falling ST-Level DEV’) were inspected in Visual Analyzer. Figures 2, 3 show averaged ERP pattern for short and long stimuli, Figures 4, 5 show averaged ERP pattern for level and falling stimuli and localization of MMN (DEV-ST) for both Word sets and Language groups. Both averaged ERP curves and head maps (right panel) indicate possible asymmetric effects between the reversed comparisons and differences between Word sets and Language groups.

To study the processing differences between duration of the vowel or falling/level pitch shape of the vowel, Word sets (‘SATA,’ ‘SAKI’), and Language groups (Estonian – EST, Russian – RUS), single trial data for respective standards and deviants covering the whole interval (–100 ms … 600 ms) were exported and further analyzed by GAMM.

### Generalized Additive Mixed Effects Model Results

For each of the four Electrodes (AF3, AF4, C3, C4) in both two Word sets (‘SATA,’ ‘SAKI’) and in four experimental setup conditions (‘Short ST-Long DEV,’ ‘Long ST-Short DEV,’ ‘Level ST-Falling DEV,’ ‘Falling ST-Level DEV’), a GAMM was fitted in R using the packages mgcv (Wood, 2006) and itsadug (Rij et al., 2020).

In order to compare the smooth curves for the Language group (EST, RUS) and Stimulus response condition (ST, DEV), a new interaction factor of these two factors was combined. The models included Group × Response (ST, DEV) as a fixed effect and smooths for time by each group condition, and a random smooth effect of the test subject. Additionally, the trial effect was tested, which improved the model despite not being significant. The models were checked and corrected for autocorrelation.

ELECTRODE∼GRxResponse+s(TIME,by=GRx

Response)+s(TRIAL)+s(SUBJECT,bs=``re”,m=1)

Due to space limits, the 32 individual model outputs are not presented in detail but can be observed from the supplementary archive^2^. The R-squared of the models ranged from 0.017 to 0.036 with the average of 0.025, e.g., the average deviance explained by the models is rather low at 2.5%, but is typical for EEG data (cf. Tremblay and Newman, 2015).

**FIGURE 6:**
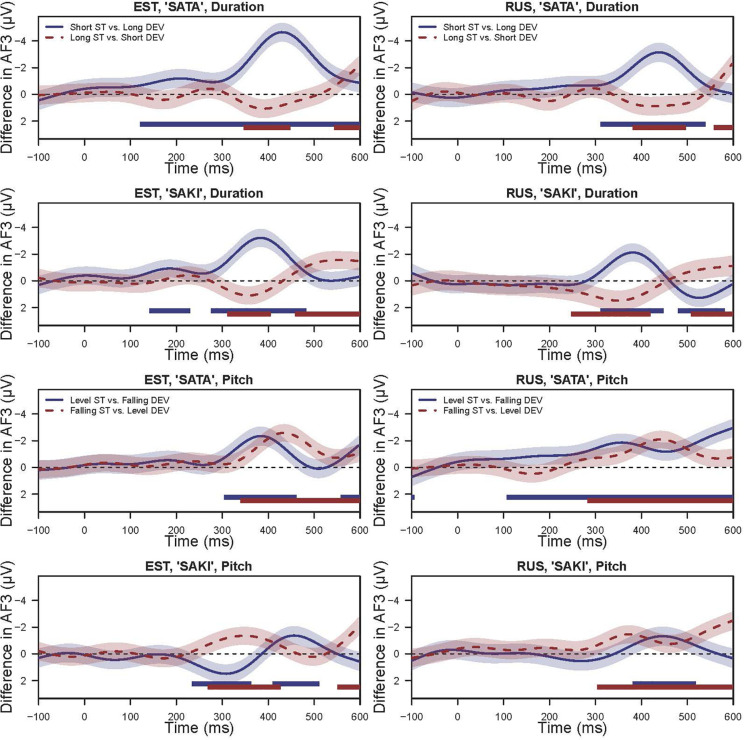
The difference (MMN) between Deviant vs. Standard indicating (a)symmetrical processing of duration (‘Short ST-Long DEV’ vs. ‘Long ST-Short DEV’) and pitch (‘Level ST-Falling DEV’ vs. ‘Falling ST-Level DEV’) within the language groups (Estonian, EST and Russian, RUS) estimated from GAMMs. Shaded area is 95% Confidence Intervals for the difference (MMN). The bold straight lines below the curves show the range where the difference is significantly different from zero. Time point 0 indicates the beginning of the initial consonant ‘s’.

Here, we will summarize these models by presenting the difference curves between the deviant and standard stimuli by Language group. As our primary focus is on the difference between the Standard vs. Deviant, we here report the *post hoc* tests results using the Wald test with Bonferroni correction, which are summarized in Table 3. While this is only a partial comparison, we also compared the differences in the smooth effects over time between the Deviant-Standard pairs. Figure 6 illustrates the results based on the AF3 (left frontal) models [figures for the other three electrodes may be found within the supplementary archive: osf.io/8uaz3/ (https://datadoi.ee/handle/33/322)]. These difference plots can also be regarded as the MMN curves. Figure 6 comprises four panels, where the comparisons of Short vs. Long conditions are on the top panels and those of Level vs. Falling conditions are in the bottom while the left and right columns are grouped by the words ‘SATA’ and ‘SAKI,’ accordingly.

**TABLE 3:** Summary of the *post hoc* comparisons of the Standard vs. Deviant stimuli within Language groups (EST/RUS if *p* < 0.05, GAMM analysis).

		AF3	AF4	C3	C4
Long-Short	‘SATA’	**EST**/RUS	**EST**/RUS	**EST**/RUS	**EST**
	‘SAKI’	**EST**	**EST**	–	**EST**
Short-Long	‘SATA’	–	–	–	–
	‘SAKI’	–	–	–	–
Level-Falling	‘SATA’	RUS	**EST**	–	**EST**
	‘SAKI’	–	–	–	–
Falling-Level	‘SATA’	**EST**	–	–	–
	‘SAKI’	RUS	**EST**	RUS	**EST**

*Est, Estonian; RUS, Russian. GAMM, Generalized Additive Mixed effects Model.*

In the case of the ‘Short ST-Long DEV’ experimental condition with the Word set ‘SATA,’ the effect was significant at *p* < 0.001 in the Estonian group in all electrodes, and in the Russian group AF3 (left frontal) at *p* = 0.011, AF4 (right frontal) at *p* = 0.020 and C3 (left temporal) at *p* = 0.009 while C4 (right temporal) was not significant. The top left panel of Figure 6 shows a strong negative peak at approximately 400 ms with slightly stronger amplitude for the Estonian group and a similar but slightly weaker amplitude for the Russian group. The same experimental condition with Word set ‘SAKI’ had less significant effects: in the Estonian group AF3 (left frontal) at *p* = 0.002, AF4 (right frontal) at *p* < 0.001 and C4 (right temporal) at *p* = 0.011, but C3 (left temporal) not significant, while in the Russian group there were no significant effects. From the top right panel of Figure 6, it can be seen that there are negative peaks for both Estonian and Russian groups similar to the left panel, but only slightly earlier and weaker.

The same stimuli in the opposite ‘Long ST-Short DEV’ condition showed no significant effects, and the upper panels of Figure 6 also show that there are no significant negative peaks in the area of interest.

In the ‘Level ST-Falling DEV’ experimental condition in the ‘SATA’ set, there was a significant effect in the Estonian group for AF4 (right frontal) and C4 (right temporal) at *p* = 0.007 while for AF3 (left frontal) and C3 (left temporal) the effect was not significant; in the Russian group the difference was only significant for AF3 at *p* < 0.001. The bottom left panel of Figure 6 shows a negative peak before 400 ms in both Language groups. In the ‘SAKI’ set there were no significant effects in either Language group, but from Figure 6 it can be seen that there is a weaker but still significant negative peak around 450 ms.

In the case of the ‘Falling ST-Level DEV’ experimental condition in ‘SATA’ set, there was only a significant effect for AF3 (left frontal) Electrode, in Estonian group at *p* = 0.021; in the Russian group there were no significant effects. From Figure 6 it can be seen that there is a negative peak around 400 ms, slightly later than in the ‘Level ST-Falling DEV’ condition. In the ‘SAKI’ set in the Estonian group there was a significant effect for AF4 (right frontal) at *p* = 0.036 and C4 (right temporal) at *p* = 0.044, while in the Russian group AF4 and C4 were not significant, but AF3 (left frontal) was at *p* = 0.018, and C3 (left temporal) at *p* = 0.003. The bottom right panel of Figure 6 shows that there is a significant negative peak between 300 and 400 ms, slightly earlier for the Estonian group when compared to the Russian group.

### Repeated Measures ANOVA Results

As GAMM analysis showed significant results, additional analyses with averaged results were conducted to look for more robust effects of asymmetric processing and differences in MMN activation. Due to small sample size and possible group differences that appeared in GAMM, the following analyses were conducted separately for both Language groups (EST, RUS).

Four time Intervals of interest (400–420, 420–440, 440–460, 460–480 ms) were included in repeated measures ANOVA and were selected through inspection of the peaks of deviant activity in each Electrode, Comparisons and Word set [single values ranged from 381.26 ms (only appeared in two peaks) to 477.44 ms] and by the results of GAMM analysis. In total 16 ANOVA models were created with 4 Comparison (‘Short ST-Long DEV,’ ‘Long ST-Short DEV,’ ‘Level ST-Falling DEV,’ ‘Falling ST-Level DEV’) × 2 Word sets (‘SATA,’ ‘SAKI’) × 2 Language groups (EST, RUS). Interval (4), Electrode (AF3, AF4, C3, C4) and Response (average activity of standard and deviant stimuli) were assigned as independent variables, and the average activity of predefined conditions as the dependent variable. The results of each comparison are reported under a given subsection.

The results are presented as pairs of comparisons of comparable stimuli (change in duration, pitch).

### Changes in the Duration of Stimuli

#### Short Standard Versus Long Deviant Stimuli

Table 4 contains the results of comparisons in the ‘Short ST-Long DEV’ experimental condition. The main effects were significant for all viewed predictors for the Estonian group in both Word sets, and for the Russian group in the Word set ‘SATA.’ Interaction Electrode × Interval was significant for Estonian group ‘SATA’ and ‘SAKI,’ and Russian group ‘SATA.’ We found no significant results in Bonferroni *post hoc* tests here. Interaction Interval × Response was significant for ‘SATA’ in both Language groups. Bonferroni *post hoc* showed significant result between the average result of each deviant and standard (MMN) in every viewed Interval for the Estonian group (*p* < 0.001, all Intervals) and Russian group (*p* = 0.043 in 400–420 ms, *p* = 0.002 in 420–440 ms, *p* < 0.001 in 440–480 ms). Interaction Interval x Response was also significant for the Russian group ‘SAKI,’ *post hoc* results were significant in 400–440 ms (*p* < 0.001). Interaction Electrode × Response was significant for the Estonian group Word set ‘SATA’ and Russian group ‘SAKI,’ *post hoc* results showed significant differences between standard and deviant stimuli in all Electrodes (*p* < 0.01) for ‘SATA’ (EST) and in AF3 (left frontal) (*p* = 0.002), C3 (left temporal) (*p* = 0.018) for ‘SAKI’ (RUS). Also, interaction Electrode × Interval × Response was significant for the Russian group Word set ‘SATA,’ and Bonferroni *post hoc* test showed significant differences between standard and deviant stimuli in Electrode C3 (left temporal) in 440–460 ms (*p* = 0.035) and in 460–480 ms (*p* = 0.008).

**TABLE 4:** ANOVA results in the ‘Short ST-Long DEV’ condition for each Language group (EST, RUS) and Word set (‘SATA,’ ‘SAKI’).

			EST						RUS					
			‘SATA’			‘SAKI’			‘SATA’			‘SAKI’		
Predictor	*df*_*Num*_	*df*_*Den*_	*F*	*P*	η^2^_*g*_	*F*	*p*	η^2^_*g*_	*F*	*p*	η^2^_*g*_	*F*	*p*	η^2^_*g*_
Electrode	3	33	**25.41**	**0.000**	**0.34**	**7.12**	**0.001**	**0.14**	**4.16**	**0.013**	**0.13**	**9.29**	**0.000**	**0.14**
Interval	1	11	**10.14**	**0.009**	**0.13**	**6.79**	**0.024**	**0.08**	**20.29**	**0.001**	**0.12**	0.30	0.593	0.00
Response	1	11	**54.63**	**0.000**	**0.66**	**65.09**	**0.000**	**0.59**	**33.34**	**0.000**	**0.46**	**4.99**	**0.047**	**0.16**
Electrode × Interval	3	33	**3.27**	**0.033**	**0.01**	**4.53**	**0.009**	**0.02**	**3.37**	**0.030**	**0.01**	1.71	0.184	0.00
Electrode × Response	3	33	**4.33**	**0.011**	**0.04**	1.46	0.245	0.03	1.98	0.136	0.03	**3.00**	**0.044**	**0.04**
Interval × Response	1	11	**5.62**	**0.037**	**0.03**	3.38	0.093	0.02	**24.96**	**0.000**	**0.07**	**21.60**	**0.001**	**0.13**
Electrode × Interval × Response	3	33	0.61	0.615	0.00	1.23	0.315	0.00	**3.19**	**0.036**	**0.00**	1.43	0.252	0.00

*Model contains average activity of Interval (400–420, 420–440, 440–460, 460–480 ms) × Electrode (AF3, AF4, C3, C4) × Response (Standard, Deviant). df_Num_ indicates degrees of freedom numerator. df_Den_ indicates degrees of freedom denominator. η^2^_g_ indicates generalized eta-squared. Significant results are marked with bold.*

To test the influence of native language upon elicited MMN, additional bootstrap analysis were performed, with 1000 resamplings separately for each Word set (‘SATA,’ ‘SAKI’) and Electrode (AF3, AF4, C3, C4) to average activation of Group × MMN. These same Intervals were included again and the activation was averaged over the Intervals (400–480 ms). Difference between Language groups appeared in Electrode AF3 (left frontal; mean difference 1.07 μV [95% CI: 0.37, 1.75]) and C4 (right temporal; mean difference 0.90 μV [95% CI: 0.35, 1.47]) for ‘SATA,’ and in C4 (right temporal; mean difference 1.46 μV [95% CI: 0.89, 2.06]) for ‘SAKI.’

See the ERP waveforms to auditory standard and deviant stimuli for both Language groups (EST, RUS) and for both Word set (‘SATA,’ ‘SAKI’) in Figure 2.

#### Long Standard Versus Short Deviant Stimuli

Table 5 contains the results of the ‘Long ST-Short DEV’ experimental condition. The main effects were significant for all viewed predictors only for the Russian group in Word set ‘SATA.’ Interaction Electrode x Interval was significant for both Word sets for the Estonian group. Interaction Interval × Response was again significant for ‘SATA’ in both Language groups. Bonferroni *post hoc* showed significant differences between the average result of each deviant and standard (MMN) in 460–480 ms for the Estonian group (*p* = 0.007) and the Russian group (*p* < 0.001). Bonferroni *post hoc* analyses for significant interactions Electrode × Interval × Response (EST ‘SATA’ and ‘SAKI’) did not show any significant interactions between standard and deviant activity in any Electrode.

**TABLE 5:** ANOVA results in the ‘Long ST-Short DEV’ condition for each Language group (EST, RUS) and Word set (‘SATA,’ ‘SAKI’).

			EST						RUS					
			‘SATA’			‘SAKI’			‘SATA’			‘SAKI’		
Predictor	*df*_*Num*_	*df*_*Den*_	*F*	*p*	η^2^_*g*_	*F*	*p*	η^2^_*g*_	*F*	*p*	η^2^_*g*_	*F*	*p*	η^2^_*g*_
Electrode	3	33	**15.10**	**0.000**	**0.25**	**8.14**	**0.000**	**0.07**	**9.19**	**0.000**	**0.20**	**4.08**	**0.014**	**0.06**
Interval	1	11	**7.80**	**0.017**	**0.09**	**5.93**	**0.033**	**0.03**	**14.07**	**0.003**	**0.09**	0.30	0.597	0.00
Response	1	11	3.75	0.079	0.14	0.02	0.877	0.00	**5.23**	**0.043**	**0.16**	0.11	0.746	0.01
Electrode × Interval	3	33	**5.66**	**0.003**	**0.01**	**4.62**	**0.008**	**0.01**	2.62	0.067	0.00	1.37	0.268	0.00
Electrode × Response	3	33	0.31	0.816	0.00	0.94	0.431	0.01	0.57	0.637	0.01	0.42	0.739	0.01
Interval × Response	1	11	**14.00**	**0.003**	**0.02**	1.88	0.198	0.01	**5.26**	**0.043**	**0.04**	3.71	0.080	0.01
Electrode × Interval × Response	3	33	**4.48**	**0.010**	**0.00**	**4.18**	**0.013**	**0.01**	1.48	0.239	0.00	1.00	0.405	0.00

*Model contains average activity of Interval (400–420, 420–440, 440–460, 460–480 ms) × Electrode (AF3, AF4, C3, C4) × Response (Standard, Deviant). df_Num_ indicates degrees of freedom numerator. df_Den_ indicates degrees of freedom denominator. η^2^_g_ indicates generalized eta-squared. Significant results are marked with bold.*

Again, similar bootstrap analysis with 1000 resamplings separately for each Word set (‘SATA,’ ‘SAKI’) and Electrode (AF3, AF4, C3, C4) was conducted to average activation of Group × MMN. The results were not significant in any comparisons.

See the ERP waveforms to auditory standard and deviant stimuli for both Language groups (EST, RUS) and for both Word sets (‘SATA,’ ‘SAKI’) in Figure 3.

#### Asymmetry in Duration: Comparisons Between ‘Short ST-Long DEV’ and ‘Long ST-Short DEV’

To check if the MMN response of two comparisons with duration (increased or decreased duration) differed from each other, a bootstrapped paired *t*-test analysis with 1000 resamplings was conducted for each Word set (‘SATA,’ ‘SAKI’), Electrode (AF3, AF4, C3, C4) and Language group (EST, RUS). The model included the average activation of Duration comparison (‘Short ST-Long DEV,’ ‘Long ST-Short DEV’) × Standard vs. Deviant difference (MMN). The results are presented in Table 6.

**TABLE 6:** Results of bootstrap analysis for ‘Short ST-Long DEV’ vs. ‘Long ST-Short DEV’ conditions with 1000 resamplings for each Word set (‘SATA,’ ‘SAKI’), Language group (EST, RUS) and Electrode (AF3, AF4, C3, C4), models contained average activation of Duration comparisons × MMN.

	EST		RUS	
	‘SATA’	‘SAKI’	‘SATA’	‘SAKI’
Electrode	Mean	Mean	Mean	Mean
AF3	–4.10 [–5.47, –2.64]*	–1.57 [–3.04, 0.18]	–3.02 [–4.55, –1.54]*	–1.25 [–2.79, 0.18]
AF4	–3.70 [–4.96, –2.64]*	–0.81 [–2.12, 0.88]	–3.19 [–4.59, –1.93]*	–0.79 [–2.48, 0.50]
C3	–4.22 [–5.76, –2.32]*	**–1.87 [–3.56, –0.16]**	–3.36 [–4.91, –1.76]*	–0.70 [–2.39, 1.20]
C4	–3.31 [–4.39, –2.13]*	**–2.04 [–3.21, –0.79]**	–2.59 [–3.73, –1.36]*	–0.12 [–1.12, 0.87]

95% CI. Significant results with p < 0.05 are marked with bold and results with p < 0.01 are additionally marked with *.

Significant asymmetric effects can be viewed for the Word set ‘SATA’ for each Electrode for both Language groups. For the Word set ‘SAKI,’ only the Estonian group had significant differences in temporal electrodes (C3, C4) but the difference is relatively small and could be random. See also Figure 6.

### Changes in the Pitch of Stimuli

#### Level Pitch Versus Falling Pitch Stimuli

Table 7 contains the results of the ‘Level ST-Falling DEV’ experimental condition. The main effects were significant for all viewed predictors only for Word set ‘SAKI’ for both Language groups. Interactions Electrode × Interval and Interval × Response were only significant for ‘SATA’ in the Estonian group. Bonferroni *post hoc* showed significant results between the average activity of each deviant and standard (MMN) for the Estonian group (*p* = 0.001 in 400–420 ms, *p* = 0.038 in 420–440 ms). Bonferroni *post hoc* analyses for significant interactions Electrode × Interval × Response (EST ‘SATA’) did not show any significant interactions between standard and deviant activity in any Electrode.

**TABLE 7:** ANOVA results in the ‘Level ST-Falling DEV’ condition for each Language group (EST, RUS) and Word set (‘SATA,’ ‘SAKI’).

			EST						RUS					
			‘SATA’			‘SAKI’			‘SATA’			‘SAKI’		
Predictor	*df*_*Num*_	*df*_*Den*_	*F*	*p*	η^2^_*g*_	*F*	*p*	η^2^_*g*_	*F*	*p*	η^2^_*g*_	*F*	*p*	η^2^_*g*_
Electrode	3	33	**9.82**	**0.000**	**0.17**	**12.93**	**0.000**	**0.22**	**6.16**	**0.002**	**0.16**	**8.64**	**0.000**	**0.14**
Interval	1	11	**39.84**	**0.000**	**0.15**	**12.29**	**0.005**	**0.10**	**5.65**	**0.037**	**0.03**	**11.35**	**0.006**	**0.04**
Response	1	11	2.54	0.139	0.09	**10.37**	**0.008**	**0.30**	0.08	0.780	0.00	**7.84**	**0.017**	**0.29**
Electrode × Interval	3	33	**5.78**	**0.003**	**0.01**	2.64	0.066	0.00	1.54	0.223	0.00	1.39	0.263	0.00
Electrode × Response	3	33	0.45	0.719	0.01	1.75	0.176	0.03	0.83	0.489	0.01	1.18	0.333	0.01
Interval × Response	1	11	**23.58**	**0.001**	**0.11**	1.63	0.228	0.00	3.08	0.107	0.00	0.11	0.751	0.00
Electrode × Interval × Response	3	33	**6.42**	**0.002**	**0.01**	1.27	0.301	0.00	0.87	0.465	0.00	0.03	0.993	0.00

*Model contains average activity of Interval (400–420, 420–440, 440–460, 460–480 ms) × Electrode (AF3, AF4, C3, C4) × Response (Standard, Deviant). df_Num_ indicates degrees of freedom numerator. df_Den_ indicates degrees of freedom denominator. η^2^_g_ indicates generalized eta-squared. Significant results are marked with bold.*

A further bootstrap analysis with 1000 resamplings separately for each Word set (‘SATA,’ ‘SAKI’) and Electrode (AF3, AF4, C3, C4) was conducted to average activation of Group × MMN, in order to compare the results between Language groups. Significant differences between Language groups results were in ‘SATA’ Electrode AF4 (right frontal) with 1.08 μV [95% CI: 0.38, 1.79], C3 (left temporal) with 0.95 μV [95% CI: 0.07, 1.92] and C4 (right temporal) with 0.97 μV [95% CI: 0.27, 1.63] for ‘SATA.’

See the ERP waveforms to auditory standard and deviant stimuli for both Language groups (EST, RUS) and for both Word sets (‘SATA,’ ‘SAKI’) in Figure 4.

#### Falling Pitch Versus Level Pitch Stimuli

Table 8 contains the results of the ‘Falling ST-Level DEV’ experimental condition. The main effects were significant for all viewed predictors only for Word set ‘SATA’ in the Russian group. Interaction Interval × Response was only significant for ‘SAKI’ in the Estonian group. Bonferroni *post hoc* showed no significant result.

**TABLE 8:** ANOVA results in the ‘Falling ST-Level DEV’ condition for each Language group (EST, RUS) and Word set (‘SATA,’ ‘SAKI’).

			EST						RUS					
			‘SATA’			‘SAKI’			‘SATA’			‘SAKI’		
Predictor	*df*_*Num*_	*df*_*Den*_	*F*	*p*	η^2^_*g*_	*F*	*p*	η^2^_*g*_	*F*	*p*	η^2^_*g*_	*F*	*p*	η^2^_*g*_
Electrode	3	33	**6.44**	**0.001**	**0.21**	**8.13**	**0.000**	**0.20**	**6.33**	**0.002**	**0.20**	**4.55**	**0.009**	**0.13**
Interval	1	11	4.39	0.060	0.04	**27.06**	**0.000**	**0.19**	**7.59**	**0.019**	**0.06**	**8.11**	**0.016**	**0.05**
Response	1	11	**22.15**	**0.001**	**0.37**	0.00	0.992	0.00	**20.29**	**0.001**	**0.36**	2.00	0.185	0.05
Electrode × Interval	3	33	1.97	0.137	0.00	1.58	0.212	0.00	2.60	0.069	0.01	0.14	0.936	0.00
Electrode × Response	3	33	2.59	0.069	0.02	0.09	0.964	0.00	1.38	0.266	0.01	1.56	0.218	0.02
Interval × Response	1	11	0.61	0.452	0.00	**8.96**	**0.012**	**0.05**	0.03	0.857	0.00	2.44	0.146	0.01
Electrode × Interval × Response	3	33	0.56	0.647	0.00	2.14	0.114	0.00	0.78	0.516	0.00	0.21	0.891	0.00

*Model contains average activity of Interval (400–420, 420–440, 440–460, 460–480 ms) × Electrode (AF3, AF4, C3, C4) × Response (Standard, Deviant). df_Num_ indicates degrees of freedom numerator. df_Den_ indicates degrees of freedom denominator. η^2^_g_ indicates generalized eta-squared. Significant results are marked with bold.*

A further bootstrap analysis with 1000 resamplings separately for each Word set (‘SATA,’ ‘SAKI’) and Electrode (AF3, AF4, C3, C4) was conducted to average activation of Group × MMN, in order to compare the results between Language groups. The only significant difference appeared in Electrode C3 (left temporal) for ‘SAKI’ with a mean difference –0.92 μV [95% CI: –1.52, –0.30].

See the ERP waveforms to auditory standard and deviant stimuli for both Language groups (EST, RUS) and for both Word sets (‘SATA,’ ‘SAKI’) in Figure 5.

#### Asymmetry in Pitch: Comparisons Between ‘Level ST-Falling DEV’ and ‘Falling ST-Level DEV’

In order to elucidate whether the MMN response of two comparisons with pitch contour (level or falling pitch) differed from each other, a bootstrapped paired *t*-test analysis with 1000 resamplings was again made for each Word set (‘SATA,’ ‘SAKI’), Electrode (AF3, AF4, C3, C4) and Language group (EST, RUS). The Model included average activation of Pitch comparison (‘Level ST-Falling DEV,’ Falling ST- Level DEV’) × Standard vs. Deviant difference (MMN). The results are presented in Table 9.

**TABLE 9:** Results of bootstrap analysis for ‘Level ST-Falling DEV’ vs. ‘Falling ST-Level DEV’ conditions with 1000 resamplings for each Word set (‘SATA,’ ‘SAKI’), Language group (EST, RUS), and Electrode (AF3, AF4, C3, C4), models contained average activation of Duration comparisons × MMN.

	EST		RUS	
	‘SATA’	‘SAKI’	‘SATA’	‘SAKI’
Electrode	Mean	Mean	Mean	Mean
AF3	1.42 [0.59, 2.38]*	**–1.75 [–2.92, –0.54]**	**1.86 [0.17, 3.83]**	–1.15 [–2.38, 0.01]
AF4	0.47 [–0.59, 1.71]	–0.96 [–1.89, 0.03]	1.87 [0.51, 3.30]*	–0.84 [–1.87, 0.24]
C3	**0.94 [0.07, 1.84]**	**–1.54 [–3.08, –0.24]**	**1.63 [0.19, 3.49]**	–0.76 [–1.84, 0.29]
C4	0.90 [–0.21, 2.16]	**–1.06 [–1.85, –0.30]**	**1.84 [0.52, 3.25]**	**–1.30 [–2.49, –0.24]**

95% CI. Significant results with p < 0.05 are marked with bold and results with p < 0.01 are additionally marked with *.

Significant asymmetry appeared for both Word sets and Language groups: for the Estonian group for ‘SATA’ in left side Electrodes (AF3, C3) and for ‘SAKI’ in Electrodes AF3, C3, C4; for the Russian group for ‘SATA’ in all Electrodes and for ‘SAKI’ C4. Again, these results should be interpreted carefully, as the differences are relatively small. See also Figure 6.

## Discussion

This study demonstrates that comparing apples to oranges is not exactly the same as comparing oranges to apples, with regards to language stimuli. Reversing the positions of standard and deviant stimuli with durational or a falling/level pitch pattern significantly changed the results of the MMN response and demonstrates clear asymmetric effects. We used the optimal MMN paradigm for presenting standard and deviant stimuli, and analyzed the data at the individual trial and at the averaged ERP level. Due to the huge number of trials in the GAMM model, this demonstrated more significant effects than the ANOVAs on averaged data with bootstrapped *post hoc* tests. The discussions and conclusions presented here are therefore based upon more conservative estimations. The only pair that elicited a clear and consistently detectable MMN was short standard and long deviant (see Figure 6 and Table 4). The same stimuli in reversed position did not show similar results; indeed the MMN activity in this comparison was hardly detectable (see Table 5). The bootstrap analysis demonstrated statistically highly significant differences between durational conditions in both language groups for ‘SATA,’ but not for ‘SAKI.’ Although duration changes did elicit predicted discrepancy between the positions of the stimuli, changes in pitch (level vs. falling) only created modest activation for both language groups. Significant differences between pitch comparisons did appear, but we need to be careful not to amplify the significant value of these results as we will discuss below. Brain activation patterns were distinctive for both language groups and stimulus words. The results only partially support our original hypotheses; nevertheless the results fit well together with previous research (Jaramillo et al., 2000; Roberts et al., 2014; Tamminen et al., 2015).

*H1: The discrimination of the deviant and standard stimuli is asymmetrical.*

The results presented here strongly agree with the sub-part elements of our first hypothesis (H1.1). A clear difference for duration change in the elicited MMN activity was discovered, providing proof to asymmetric effects in processing of linguistic stimuli: the deviant with increased duration created a significant MMN response for both language groups, while the deviant with decreased duration failed to produce the MMN. This is in accordance with previous studies showing that the increase of the duration is easier to notice than the decrease (Jaramillo et al., 2000; Jacobsen and Schröger, 2001; Takegata et al., 2008; Colin et al., 2009; Peter et al., 2010). Our results for durational differences clearly support the feature-detector hypothesis (Bishop et al., 2005; Timm et al., 2011) being in concordance with the underspecification of phonetic features (Eulitz and Lahiri, 2004; Politzer-Ahles et al., 2016), and contradict the information-content hypothesis (Sinkkonen et al., 1996).

The stimuli with pitch change (H1.2) did not elicit a consistent and significant MMN (see Figure 6 and Tables 7, 8). The GAMM analysis indicates that for pitch, there is something symmetry-like for both Estonian and Russian language groups. Further ANOVAs with averaged data reduced the differences between processing of standard and deviant stimuli considerably (Table 7). Bootstrapping analysis did show significant differences between pitch comparisons, but compared to the duration comparisons for ‘SATA,’ these differences are smaller (95% CIs very close to 0). As the MMN responses for pitch comparisons were very small and non-systematic (due to small sample size and issues with pitch stimuli that we will describe further below), we refrain from presenting the differences as a proof for asymmetry. Likewise, no sufficient evidence for underspecification (Eulitz and Lahiri, 2004), feature-detector (Bishop et al., 2005; Timm et al., 2011) or information-content hypothesis (Sinkkonen et al., 1996) were found. Therefore, we did not find strong support for the second part of the first hypothesis (H1.2).

Regarding underspecification, the results related to H1 suggest that the temporal features are specified in the Estonian quantity model while the tonal features may be underspecified.

*H2: The asymmetrical discrimination between the deviant and standard language stimuli depends on long-term language experience (native language).*

According to the feature-detector framework, we expected that for something that is common in one’s native language and has become a stronger feature due to long-term language experience, the deviance of this feature results in a larger MMN. Specifically, we expected that, as the duration and pitch contour are important features in the Estonian language (able to change the word meaning), the MMNs for Estonians are larger in amplitude than for Russians in both comparisons. However, we got a consistently stronger pattern of MMNs for Estonians than for Russians in both Duration comparisons (‘Long ST-Short DEV’ and ‘Short ST-Long DEV,’ see Figure 6) but the results for pitch comparisons were weak (‘Level ST-Falling DEV’ and ‘Falling ST-Level DEV,’ see Figure 6). Similar logic can be extended to difference in processing ‘SATA’ and ‘SAKI,’ too: more frequent (i.e., more familiar) ‘SATA’ results in greater MMN.

Hypothesis 2.1 found partial support – we expected to see asymmetry for both comparisons, duration and pitch, in Estonians, though the results suggest strong asymmetry for duration only. Again, a small sample size could be one explanation as to why the comparisons with level and falling pitch did not result in a consistent MMN; though as the comparisons with duration changes provided solid MMN results, the sample size cannot be the only reason. Perception of pitch could be a more complex process than detecting durational differences, or durational information could be analyzed first on the neurobiological level. Although previous research (Näätänen et al., 1989) has asserted that the reversed design has proven itself and shows comparable results, it might not be suitable for more complex linguistic processes if the manipulation indeed includes more than simple sounds.

The results of the Russian native speakers rather supported hypothesis H2.2 (i.e., Russian native speakers show an asymmetry in the MMN only in the duration comparison). However, this might be misleading as the comparisons with pitch did not provide strong results for either language group. It would be wrong to assume that the absence of MMN activity for pitch stimuli among Russian speakers would show clear differences with Estonian native speakers in their ability to sense pitch. Politzer-Ahles et al. (2016) also found similar results for both: native speakers of Mandarin and non-Mandarin speaking participants displayed no difference in sensitivity to tone contrast between language groups, despite different language backgrounds. They explained it with acoustic processing of the tone which, extends previously made claims that tonal features can carry different (acoustic instead of linguistic) meaning for non-native speakers (Gandour et al., 2000; Chen et al., 2015). However, another explanation might be connected with word stress in Russian, which can – in some conditions – work similarly to pitch (Jones and Ward, 1969; Bondarko, 1977).

### Possible Explanations for the Results

#### Stimulus Words

We used linguistic stimuli that are highly similar to real words, which raises the ecological validity of the study. The stimuli were chosen to represent the features –pitch and duration – that allow making a decision about the meaning of the word in Estonian language (Lippus et al., 2013). Short duration and level pitch could be considered as “default” features in Estonian, meaning that when a standard (in our study, the stimulus with short and level first vowel, see Figure 1) is a more prototypical representative of a phonological category than a deviant, bigger MMN amplitudes are expected (Ikeda et al., 2002). However, there were possibly some problems with the selection of the stimuli. Linguistic stimuli, especially when these are highly similar to natural speech, incorporate many different features at the same time and it is not possible to distinguish the processing of every feature separately. This shows the enormous effort our brain has to make to process sounds and create an understandable meaning out of it.

The language groups did have some differences in perception of linguistic stimuli, but these were rather small as illustrated in Figures 2–5. Relatively similar activation patterns could be influenced by the structural form of the stimuli or perhaps the Russian native speakers found connections between the used (Estonian language based) stimuli and some of their own meaningful words. Instead, the activation patterns of the brain differed more than previously expected between the used stimuli words. The word set ‘SATA’ elicited slightly later MMN compared to ‘SAKI,’ and the differences were even more apparent for the Russian-native participants. Both word sets were chosen carefully so that each form would have a meaning in Estonian and still be as structurally similar to each other as possible [beginning (‘SA’), one plosive consonant (‘T’/’K’) and vowel (‘A’/I’) in the end]. One possible source for the differences between the stimuli words could be the familiarity of words. All forms of the word set ‘SATA’ are well known and more common in Estonian compared to ‘SAKI.’ We wanted to use word sets that have meaning in all three Estonian quantities, but the inevitable issue with using meaningful words is that it is difficult to find structurally similar words that would also be used with similar frequency. As noted in the introduction, words that have fewer repetitions (i.e., are less common) can produce an MMN with a smaller amplitude compared to well-known words (Aleksandrov et al., 2017a). Then again, different word frequencies are natural for real-life communication, leading us to conclude that expanded word sets should be included into future studies in order to better elucidate these aspects of the approach.

#### Coarticulation

One possible explanation for differences between stimulus words could be coarticulation, where one phoneme can influence how the previous sounds were perceived (Grosvald, 2009). Syllables ‘TA’ and ‘KI’ might have changed how the previous syllable ‘SA’ was perceived (Magen, 1997; Grosvald, 2009). While the stimulus set ‘SATA’ was chosen to provide comparable results with previous research (Lippus et al., 2009) and the other sets were created to represent similar features, the extent of possible coarticulation effects needs more exploration. Altogether, the pattern warns us to take the meaning of stimuli into consideration when drawing conclusions about processing of stimuli or designing comparisons for MMN.

#### Magnitude of Change

Joutsiniemi et al. (1998) have previously discussed some possible issues with the decrease of the deviant as the magnitude of the change can be important – the decrease has to be at least 50% of the duration of the standard to be able to elicit a significant MMN. Considering this, the current results were predictable, as in our comparison the decrease of the duration of deviant stimulus was considerably less than that (20.7%, 290 versus 170 ms in the vowel with the feature manipulation) but it is explainable by the use of natural-like linguistic stimuli. Also, the magnitude of the pitch change was even smaller (not easy enough to distinguish the stimuli from each other) (Jaramillo et al., 2000).

#### Lateralization

Here, we analyzed the results from electrodes that capture the signals of brain areas (frontal, temporal) that have been previously found to be most closely connected with auditory processing (Deouell, 2007; Dürschmid et al., 2016): left and right frontal and temporal locations. This allowed us to additionally look for possible lateralization effects. The right temporal lobe has been previously associated with prosodic (intonation, stress, rhythm) and acoustic (fundamental frequency) attributes, while the left side is responsible for substantial processing related to phonetics and the meaning of a perceived sound (Shestakova et al., 2002; McGettigan et al., 2012; Kreitewolf et al., 2014). For example, lateralization differences have been found between tonal (Mandarin Chinese) and non-tonal (English) languages in a discrimination task of pitch patterns using Mandarin words (Klein et al., 2001). In contrast to the activation of the right hemisphere for English speakers, Mandarin speakers showed the activation of the left, suggesting that the absence of tone in one language could alter the way in which pitch information is processed. In our study, the lateralization of MMN activity showed some possible activation differences (Tables 6, 9) though the results are inconclusive. Still, we cannot rule out some lateralization effects influencing the results.

### Limitations and Strengths

Throughout the course of this examination, we have identified a number of areas where future studies could build upon the work presented here. The number of participants was not representative enough for solid conclusions. The study, however, still demonstrates significant effects of perceptual asymmetries even with 12 + 12 participants in one comparison (‘Short ST-Long DEV’ and ‘Long ST-Short DEV’). In principle, more stimuli words would also need to be incorporated into the experimental design in order to have a better overview of influential effects – meaning vs. pseudo word vs. noise, coarticulation, underspecification and language background. However, while the variation in word sets was hypothesized to be a strength through greater generalizability, in practice it appears to create extra variability, necessitating additional analysis in future studies.

It can be difficult to clearly distinguish lower order auditory processing (reflected by the N1 component of the ERPs) from higher order processes in auditory deviance detection (MMN) (Takasago et al., 2020). One inescapable complication with a reversed design MMN is that a deviant stimulus with its fresh units always elicits a stronger N1 than a standard stimulus. This can be avoided by employing an equiprobable control condition (e.g., Schröger and Wolff, 1996), or reduced by using the optimum paradigm (Näätänen et al., 2004), which was done here. In the optimum design, the number of standards is considerably lower – 1:3 in our case versus, for example, 1:9, 1:8, or 1:7 in a traditional oddball design – and thus, the difference between N1s due to refractory attenuation is smaller. Also, the actual time-range of the difference that is 130–250 ms since the start of the physical difference for duration condition supports that N1 alone is not the complicating factor, as we would expect N1 to appear rather earlier than MMN (Näätänen et al., 2005). The results show that physically the same difference in duration or pitch is processed differently due to previous language experience and language-specific memory traces. This can support the contribution of MMN in such comparisons, showing how the previous experience helps to build a model of sensory input further shaping the lens through which the world is perceived.

Despite this, we maintain that this approach yields numerous advantages as well: Firstly, through the utilization of stimuli that represent real words, not only artificial ones. Second, for duration and pitch, symmetrical comparisons (with real-word stimuli) were used. Lastly, participants came from two highly different language groups, encouraging the use of similar designs for targeted comparisons, with participants from different language groups searching for universal patterns.

To conclude, auditory perceptual asymmetry is highly dependent of the language background and the specific features within a language. The study we present here is the first to explore auditory perceptional asymmetry through the lens of the Estonian language and providing a cross-linguistic comparison with structurally different Russian language, expanding the generalizability within the field.

## Data Availability Statement

The raw data supporting the conclusions of this article will be made available by the authors, without undue reservation. The data is available at osf.io/8uaz3/ (https://datadoi.ee/handle/33/322).

## Ethics Statement

The studies involving human participants were reviewed and approved by Research Ethics Committee of the University of Tartu. The patients/participants provided their written informed consent to participate in this study.

## Author Contributions

KK, NP, and LK developed the study, set the theoretical framework, selected the methodology, and formulated the hypotheses. PL created the stimuli. LK conducted the experiments and wrote the first draft of the manuscript. LK and PL ran the data analyses. All the authors edited the manuscript and have approved the final version.

## Conflict of Interest

The authors declare that the research was conducted in the absence of any commercial or financial relationships that could be construed as a potential conflict of interest.

## Footnotes

1 www.cl.ut.ee/ressursid/sagedused/

2 osf.io/8uaz3/ (https://datadoi.ee/handle/33/322)
